# Twist-Angle-Dependent Electrocatalytic Activation of Basal Plane MoS_2_ in Twisted Bilayer Structures

**DOI:** 10.3390/ma19153211

**Published:** 2026-07-28

**Authors:** Yuhang Chen, Huanbo Zhang, Hang Lu, Xiongfeng Li, Zhenyao Huang, Mengyu Yan

**Affiliations:** 1 State Key Laboratory of Advanced Technology for Materials Synthesis and Processing, School of Materials Science and Engineering, Wuhan University of Technology, Wuhan 430070, China; cyh344767@whut.edu.cn (Y.C.); whutzhb2201@whut.edu.cn (H.Z.); 348935@whut.edu.cn (H.L.); 297062@whut.edu.cn (X.L.); a2328881382@163.com (Z.H.); 2 Foshan Xianhu Laboratory, Xianhu Hydrogen Valley, Foshan 528200, China

**Keywords:** twist angle engineering, nano device, hydrogen evolution reaction, molybdenum disulfide

## Abstract

The electrocatalytic performance of transition metal dichalcogenides (TMDs) is often hindered by their basal planes. Although defect engineering and heteroatom doping are employed, improving the electrocatalytic activity of the basal plane without disrupting the in-plane lattice structure remains a challenge. The interlayer twist serves as an effective strategy to preserve the in-plane lattice integrity of TMDs and tune their electrocatalytic performance. In this work, high-quality twisted bilayer MoS_2_ (TBL-MoS_2_) was fabricated through polydimethylsiloxane (PDMS)-assisted mechanical exfoliation and dry transfer stacking. On-chip electrochemical nanodevices were subsequently constructed to investigate hydrogen evolution reaction (HER) performance at different twist angles. This demonstrates that bilayer MoS_2_ with a twist angle of 15° (TBL-15°) exhibits superior hydrogen evolution reaction performance with a Tafel slope of 63 mV/dec, significantly lower than the value of 202 mV/dec of the pristine bilayer. This work paves the way for twist angle engineering in designing high-performance 2D electrocatalysts.

## 1. Introduction

Hydrogen is a clean and sustainable energy carrier. It is increasingly recognized as essential for decarbonizing heavy industry, long-haul transportation, and energy storage [1]. Green hydrogen is produced via renewable-powered water electrolysis. The hydrogen evolution reaction (HER) is the cathodic half-reaction of this process. Green hydrogen offers a pathway to carbon neutrality and energy autonomy [2]. Conventional hydrogen is produced from fossil fuels. The widespread adoption of green hydrogen is constrained by the high cost and scarcity of Pt-based catalysts [3]. Therefore, affordable and high-performance alternatives are urgently needed. Transition metal dichalcogenides (TMDs), particularly MoS_2_, are extensively studied as electrocatalysts for HER [4]. However, the basal plane of MoS_2_ generally shows lower electrocatalytic activity than its edge sites [5,6,7]. To address this limitation, conventional engineering strategies such as heteroatom doping [8], vacancy generation [9], phase transitions [10], and strain engineering [11] have been extensively adopted to enhance in-plane electrocatalytic activity. These methods primarily rely on modulating the local electronic structure [12,13]. Nevertheless, these approaches often degrade lattice perfection and material purity. Therefore, developing a strategy to modulate the electronic structure without chemical modification remains desirable. Twist angle engineering is a potential candidate that can activate the basal plane [14]. Recent work has also shown that twistronic can directly affect interfacial electrochemistry [15,16,17,18,19]. Bediako and co-workers found that heterogeneous electron transfer kinetics at twisted bilayer graphene electrodes strongly depend on the twist angle, especially near the magic angle [14,20]. Despite these advances in graphene, the twist-angle-dependent HER performance of semiconducting TMDs such as MoS_2_ remains largely unexplored.

Reliable investigation of twist-angle-dependent electrocatalytic behavior relies on high-quality two-dimensional (2D) materials. Crystal defects, surface contamination, interfacial residues, wrinkles, and local strain can alter charge-transfer pathways and modify the intrinsic catalytic response [21]. Interfacial nonuniformity may further affect interlayer coupling and electronic reconstruction, leading to significant variations in HER activity [22]. High crystal quality and clean interfaces are therefore essential for establishing a direct correlation between twist angle and catalytic performance [23].

Preparation methods for 2D materials can be classified into exfoliation and growth approaches [24]. Chemical vapor deposition and molecular beam epitaxy offer advantages for scalable device fabrication. Growth-induced defects, grain boundaries, substrate interactions, and structural inhomogeneity remain difficult to eliminate completely [25,26]. These factors can obscure intrinsic interlayer-coupling effects and introduce uncertainties in catalytic measurements. Mechanical exfoliation is an effective route to obtain high-quality 2D materials [27]. The original crystal structure can be preserved during the exfoliation process. Low defect density, atomically flat surfaces, and excellent crystallinity can therefore be achieved. Such characteristics make mechanically exfoliated flakes particularly suitable for studies focused on intrinsic physical and chemical properties.

Construction of twisted bilayer structures further requires precise transfer and stacking techniques. Dry-transfer methods enable the accurate control of layer alignment and minimize structural damage [28]. Precise control of stacking configuration is also essential for achieving reproducible twist-angle-dependent properties. Therefore, the precise fabrication of twisted MoS_2_ structures is essential for understanding the relationship between interlayer coupling and HER activity.

In this study, we aim to systematically investigate the influence of twist angle on the electrocatalytic HER performance of TBL-MoS_2_. We used polydimethylsiloxane (PDMS)-assisted mechanical exfoliation to obtain high-quality monolayer MoS_2_ with lateral dimensions exceeding 50 μm. Twisted bilayer MoS_2_ structures were subsequently assembled through the dry transfer method. Electron beam (e-beam) lithography was used to confine the electrochemical reaction area to the twisted overlap region. Enhanced electrocatalytic activity was observed near a twist angle of 15°. TBL-15° MoS_2_ exhibited an overpotential of 182 mV at a current density of 10 mA/cm^2^ and a Tafel slope of 63 mV/dec. This work provides experimental evidence for the role of moiré superlattice effects in interfacial electrochemistry and offers a chemical modification-free strategy for designing high-performance TMDs electrocatalysts.

## 2. Materials and Methods

### 2.1. Device Fabrication

**Figure 1:**
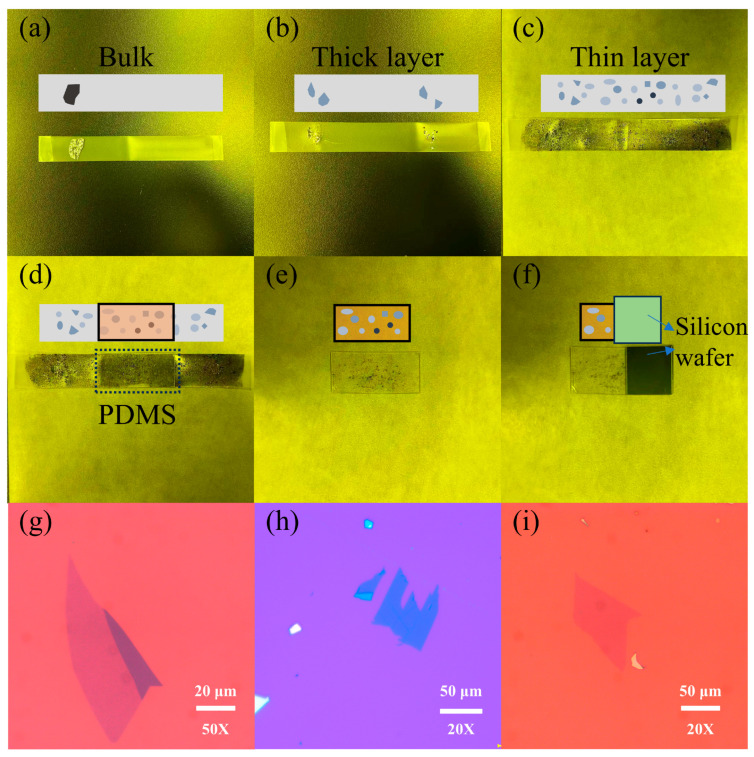
(**a**–**f**) The process of the PDMS-assisted mechanical exfoliation method. Insets showed process diagram. (**g**–**i**) Optical images of Monolayer MoS_2_.

First, a small amount of thick-layer MoS_2_ was picked up from a bulk crystal using a dedicated adhesive tape for mechanical exfoliation (Figure 1a,b). The tape was then repeatedly folded to disperse the material as uniformly as possible on its surface (Figure 1c). Subsequently, a PDMS stamp was placed onto the tape and gently pressed (Figure 1d) to ensure full contact with the material. The PDMS stamp was then lifted off (Figure 1e). Afterwards, a cleaned SiO_2_/Si substrate was brought into contact with the PDMS stamp, and an appropriate pressure was applied to transfer the material onto the substrate surface (Figure 1f). In addition to mechanical pressing for transfer, PDMS also exhibits good thermal release properties. The stamp was horizontally attached to a clean silicon wafer. Heating to 80 °C for 5 min could release the material onto the silicon wafer. Monolayer MoS_2_ with lateral dimensions exceeding 50 μm was produced by the exfoliation method (Figure 1g–i). Larger flakes improved the efficiency of sample screening. They also provided more sufficient operating space for subsequent rotation, stacking, and device fabrication.

**Figure 2:**
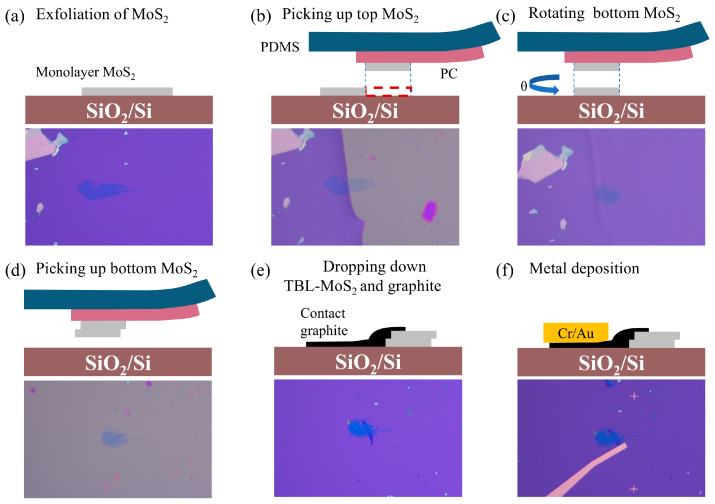
(**a**–**f**) Schematic illustration of the device fabrication process.

A 12 wt% polycarbonate (PC) solution was prepared using dichloromethane (CH_2_Cl_2_) as the solvent. The solution was dropped onto a glass slide and spread with a scraper to form a thin PC film. We fixed the film onto a PDMS stamp mounted on a glass slide. The glass slide was fixed on a rotatable holder of the transfer stage, and the target sample was placed on a heated rotatable stage (Figure 2a). The stage temperature was set to 120 °C. The glass slide was lowered until the PC film contacted half of the sample (Figure 2b) and then raised by 500 µm (Figure 2c). The desired twist angle was adjusted by rotating the stage. Alignment was carried out under an optical microscope. The displacement axis was then lowered to establish contact (Figure 2d). The temperature was increased to 170 °C to release the PC film onto the sample. After cooling to room temperature, the silicon wafer was immersed in CH_2_Cl_2_ for 5 min to remove the PC layer completely. A twisted MoS_2_ structure was thus obtained. The same procedure was used to transfer a thin graphite flake onto the edge of the twisted MoS_2_ structure, thereby improving the electrical contact (Figure 2e). Finally, ultraviolet photolithography was employed to define the electrode patterns, followed by the thermal evaporation of a 5 nm Cr layer and a 25 nm Au layer (Figure 2f). A 2 μm Polymethyl methacrylate (PMMA) insulating layer was spin-coated onto the silicon wafer after metal electrode deposition. Electron beam lithography was used to define the electrochemical reaction area.

### 2.2. Materials Characterizations

AFM morphology was characterized using Dimension IconIR (Bruker, Bilerika, MA, USA). Second harmonic generation (SHG) signals were obtained using an MStarter 100-SHG confocal microscope system (Nanjing Metatest Optoelectronic Technology Co., Ltd., Nanjing, China). A femtosecond laser (140 fs, 80 MHz) with an excitation wavelength of 800 nm was coupled into the microscope optical path and focused onto the sample surface through a 40× objective lens. The TEM images were obtained using a Themis Z transmission electron microscope with an Image Corrector (Thermo Fisher, Waltham, MA, USA). The Raman and photoluminescence (PL) spectra were obtained using the HORIBA HR EVO Raman system (HORIBA, Kyoto, Japan) with an excitation laser of 532 nm. Electrochemical catalysis and quantum capacitance measurements were performed using an Autolab PGSTAT100N electrochemical workstation (Metrohm, Herisau, Switzerland) combined with a TTPX cryogenic probe station (Lake Shore, Westerville, OH, USA). Current–voltage (I-V) characteristics were recorded using a B1500A semiconductor device parameter analyzer (Keysight Technologies, Santa Rosa, CA, USA) under ambient conditions. In the I-V tests, a probe station with two external electrodes spaced 4 mm apart was used to contact the sample. The I-V measurements were performed at 25 °C in ambient air. The applied voltage was varied between −0.1 V and +0.1 V, and the resulting current was recorded at each step. The resistance was then calculated using Ohm’s law:

(1) $$R=\frac{V}{I},$$

where *V* was the applied voltage, and *I* was the measured current. This allowed for the determination of the resistance at different potentials and provided insight into the material’s electronic properties.

### 2.3. Electrochemical Test

The electrochemical performance test was conducted using the standard three-electrode system. A commercial carbon rod and the TBL-MoS_2_ were used as the counter and working electrodes, respectively. A saturated calomel electrode (SCE) was used as the reference electrode for the electrocatalytic test. The electrolyte was 0.5 mol/L H_2_SO_4_. The electrochemical test was conducted at 25 °C and normal pressure in ambient air. Cyclic voltammetry (CV) was used for the activation process, with a scan rate of 5 mV/s, until the CV curve did not change significantly compared to the previous cycle. The CV scans started from open circuit potential (OCP, ~0.1 V vs. SCE) and negatively scanned to −0.8 V vs. SCE. Afterwards, the CV scanned positive to 0.2 V vs. SCE. Linear sweep voltammetry (LSV) was performed by sweeping the potential negatively from the OCP (~0.1 V vs. SCE) to −0.8 V vs. SCE. Three independently fabricated devices were measured for each sample group, and the error bars represent the standard deviation. All reported overpotentials were converted using the calibrated reference electrode offset without further IR compensation. The specific details are contained in the Supplementary Material (Figure S1).

## 3. Results and Discussion

**Figure 3:**
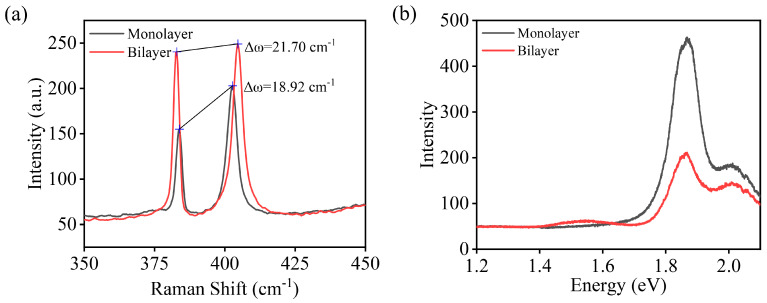
(**a**) Raman spectra and (**b**) PL spectra of mechanically exfoliated Monolayer and Bilayer MoS_2_.

The layer dependence of optical properties allows for the precise identification of the atomic layer thickness of MoS_2_. As illustrated in Figure 3a, the Raman spectrum of monolayer MoS_2_ reveals a low frequency difference of 18.92 cm^−1^. In the bilayer region, the increased resilience of inter-layer van der Waals interactions results in greater peak separation. Correspondingly, the PL spectra in Figure 3b display a sharp contrast in emission intensity. The monolayer region, characterized by a direct band gap induced by quantum confinement, exhibits intense photoluminescence. In contrast, the PL intensity in the bilayer region is significantly quenched, and the emergence of indirect exciton peaks indicates a transition from a direct to an indirect band gap [29].

**Figure 4:**
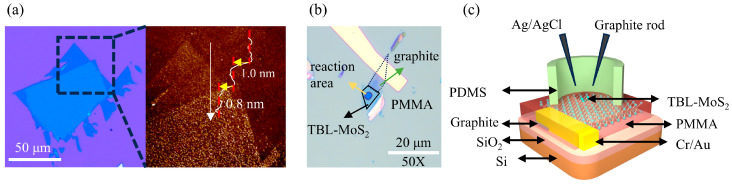
(**a**) Optical microscope and AFM images of the TBL-15° MoS_2_. (**b**) The optical image of the reaction window exposed by e-beam lithography. (**c**) Schematic diagram of the device structure.

High-quality TBL-MoS_2_ samples for electrochemical testing were fabricated using the dry transfer method, yielding clean interfaces, as confirmed by optical and AFM images (Figure 4a). The thicknesses of the monolayer MoS_2_ and the TBL-MoS_2_ were measured to be 1.0 nm and 1.8 nm, respectively. The electrochemically active region was defined by an e-beam lithography process, which etched a reaction window exposing the TBL-MoS_2_ electrode to the electrolyte (Figure 4b). The exposed reaction area was determined from the calibrated optical microscopy images. The exposed areas of the TBL-MoS_2_ used in this work were in the range of 20–50 μm^2^. Finally, PDMS was employed to separate the reaction area from the external electrodes, yielding an on-chip electrochemical nanodevice (Figure 4c). TBL-MoS_2_, graphite rod, and an Ag/AgCl electrode served as the working electrode, counter electrode, and reference electrode, respectively.

**Figure 5:**
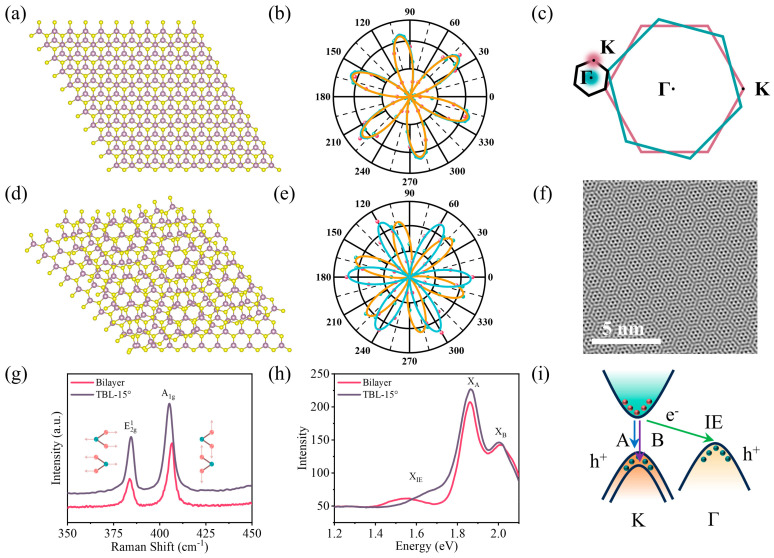
(**a**) Top view of the atomic structure of bilayer MoS_2_. (**b**) Polar plots of parallel components of SHG in bilayer MoS_2_. (**c**) Brillouin zones of the TBL-15° MoS_2_ (black hexagons) and its component monolayers (red and green hexagons). (**d**) Top view of the atomic structure of TBL-15° MoS_2_. (**e**) Polar plots of parallel components of SHG in TBL-15° MoS_2_. (**f**) TEM image of TBL-15° MoS_2_. (**g**,**h**) Raman and PL spectra of bilayer and TBL-15° MoS_2_. (**i**) The PL emission process between the conduction band and the valence band in bilayer and TBL-15° MoS_2_.

The structural and optical characterization was performed to investigate the angle-dependent microstructure. The bilayer and TBL-15° MoS_2_ were employed to fabricate the nanodevices (Figure 5a,d). Polarization-resolved SHG was utilized for the accurate determination of the twist angle [30]. Monolayer MoS_2_ exhibited a characteristic sixfold symmetry feature (Figure 5b,e) [31]. The phase difference of SHG was used to calculate twist angles in TBL-MoS_2_. The bilayer and TBL-15° MoS_2_ showed a twist angle of 0.32° and 15.36°, respectively, with an uncertainty error of ±0.4°. The SHG signals of the TBL-14° and TBL-16° MoS_2_ are shown in Figure S2. Additionally, the spherical aberration corrected transmission electron microscope (AC-TEM) image clearly showed the Moiré pattern in the TBL-15° MoS_2_ (Figure 5f). The measured period length (L) was approximately 1.171 nm. According to the Moiré periodic formula (Figure S3a) [32],

(2) $$L\approx \frac{a}{2\sin\left(\frac{\theta }{2}\right)},$$

in which a is the lattice constant, and θ is the twist angle. The corresponding twist angle was 15.48°, highly consistent with the SHG results. The SAED patterns provided further evidence for this conclusion. The well-defined and symmetric diffraction spots indicated that no significant lattice disorder or polycrystalline domains were present within the selected area (Figure S3b).

The formation of the Moiré superlattice fundamentally alters the electronic structure [33,34]. In reciprocal space, the Brillouin zones of the two twisted MoS_2_ overlap with a twisted offset. This misalignment caused the original bands to fold into a mini-Brillouin zone (Figure 5c). The structural changes were reflected in distinct optical and vibrational signatures. Figure 5g presents the Raman spectra of bilayer MoS_2_ and TBL-15° MoS_2_. These spectra covered the range from 350 cm^−1^ to 450 cm^−1^. The frequency difference (Δω) between the two characteristic modes decreased as the twist angle increased. The bilayer MoS_2_ showed peaks at 383.83 cm^−1^ ($E_{2g}^{1}$) and 406.59 cm^−1^ (A_1g_), corresponding to a Δω of 22.76 cm^−1^. However, the TBL-15° MoS_2_ demonstrated a smaller separation of 20.77 cm^−1^, with peak positions at 384.45 cm^−1^ ($E_{2g}^{1}$) and 405.22 cm^−1^ (A_1g_). The redshift of the A_1g_ peak is due to reduced interlayer interactions. Conversely, the change in the vibration frequency of the $E_{2g}^{1}$ peak is attributed to an increase in long-range Coulombic interactions among Mo atoms [35].

The PL spectrum based on the twist angle was depicted in Figure 5h. The PL spectra of bilayer and TBL-15° MoS_2_ can be fitted into three distinct peaks. These peaks correspond to the A-exciton (X_A_), the B-exciton (X_B_), and the indirect exciton (X_IE_) [36]. Figure 5i illustrates their respective excitation processes. The spin–orbit coupling splits the degenerate K point energy level into spin-up and spin-down subbands [37]. The X_A_ arose from a direct transition between the conduction band minimum (CBM) and the valence band maximum (VBM) at the K point. The X_B_ resulted from a transition between the CBM and the lower spin-split valence band. The X_IE_ originated from a transition between the VBM at the Γ point and the CBM at the K point. The X_A_ peak remained nearly unchanged at 1.86 eV as the twist angle increased to 15°. This stability indicated the insensitivity of the X_A_ to twist angle variations [38]. Conversely, the X_IE_ peak shifted from 1.55 eV to 1.67 eV. This shift confirmed band renormalization and a strong dependence on the twist angle [39,40,41].

**Figure 6:**
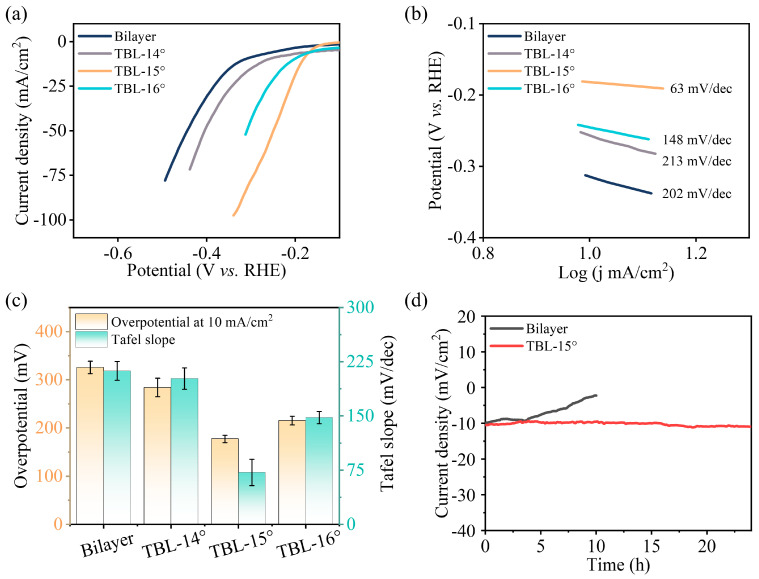
(**a**) Linear sweep curves of MoS_2_ (bilayer, TBL-14°, TBL-15°, and TBL-16°). (**b**) The corresponding Tafel curves of the polarization curves previously depicted in Figure 3a. (**c**) Summary of overpotential at 10 mA/cm^2^ and Tafel slopes with error bars from repeated measurements. (**d**) The electrocatalytic durability test of the TBL-15° MoS_2_.

Optical and structural characterizations confirmed the precise control of the twist angle in the MoS_2_. Following this validation, the HER activity was evaluated in a standard three-electrode configuration. All samples were fabricated under the same conditions. Continuous CVs were used to electrochemically activate the samples before testing (Figure S4). The LSV tests were conducted at a sweep speed of 5 mV/s (Figure 6a). Three independent devices were tested for every twist angle (Figure S5). Reaction kinetics were further evaluated by analyzing the Tafel slopes obtained from the LSV curves (Figure 6b). The Tafel slopes were 202, 213, and 148 mV/dec for the bilayer, TBL-14°, and TBL-16° MoS_2_, respectively. These values exceeded 120 mV/dec for the classic Volmer-dominated reaction pathway, indicating that polarization was influenced beyond intrinsic charge-transfer kinetics. The Tafel was also influenced by the contacting resistance between the electrode and catalysts, as well as the resistance of the MoS_2_. For the TBL-15° MoS_2_, the Tafel slope was 63 mV/dec, lying between the ideal Heyrovsky and Volmer limits. This indicated that the HER was not determined solely by a single hydrogen adsorption or desorption process, but was combined with the influence of resistance. TBL-15° MoS_2_ required an overpotential at 10 mA/cm^2^ (η_10_) of 182 mV vs. RHE (Figure 6c). This value was significantly lower than the other samples. Specifically, the TBL-16°, TBL-14°, and bilayer MoS_2_ showed overpotentials of 222, 262, and 322 mV vs. RHE, respectively. The decrease in the Tafel slope and overpotential indicated that the introduction of the twist angle significantly enhanced the electrocatalytic activity. The TBL-15° MoS_2_ exhibited competitive electrocatalytic performance relative to recently reported MoS_2_ electrocatalytic nanodevices (Figure S6) [42]. Chronoamperometry was used to evaluate long-term stability. The electrocatalytic durability test was conducted under 25 °C and normal pressure conditions. After the material was activated using cyclic voltammetry, the overpotential η required at a current density of 10 mA/cm^2^ was calculated. Then, the voltage was set to η_10_, and the long-term electrocatalytic stability of the material was tested. The TBL-15° MoS_2_ maintained robust performance over 24 h (Figure 6d). At the 16th hour of the test, 2 μL of 0.5 mol/L H_2_SO_4_ was added, resulting in a slight increase in the current density. Bilayer MoS_2_ showed significant performance degradation during the 10 h stability test, with the current density decreasing by 77.5%.

**Figure 7:**
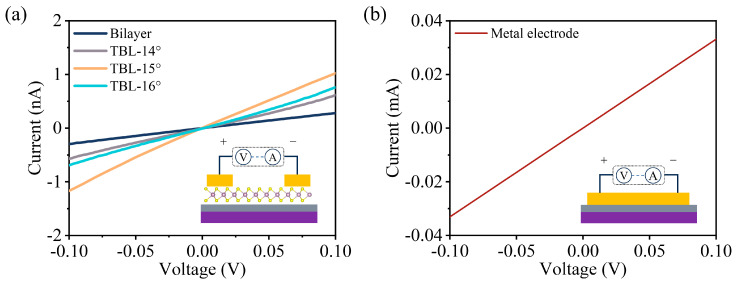
(**a**) I-V curves measured for bilayer, TBL-14°, TBL-15°, and TBL-16° MoS_2_. Insets showed the measurement geometry for the TBL-MoS_2_ devices. (**b**) I-V curves measured for the metal electrode. Insets showed the measurement geometry for the metal electrode.

I-V measurements were performed to assess the electrical performance, as variations in the electronic structure directly influence charge transport behavior. Relative to the bilayer MoS_2_ (3.47 × 10^8^  Ω), all twisted configurations demonstrated lower electrical resistance. The TBL-14° and TBL-16° MoS_2_ yielded resistances of 1.69 × 10^8^ Ω  and 1.38 × 10^8^ Ω, respectively (Figure 7a). Notably, the TBL-15° MoS_2_ achieved the lowest resistance of 9.13 × 10^7^  Ω. Under the same testing conditions, the resistance of the metal electrode was 3.01 × 10^3^ Ω (Figure 7b). These results confirmed that the twist angle promotes electron transport, thereby increasing the overall conductivity.

## 4. Conclusions

This study demonstrates a strategy to activate the basal plane of MoS_2_ through twist angle engineering. Constructing twisted bilayer MoS_2_ within the angle range of 14–16° results in significantly enhanced electrocatalytic performance of the HER. The resulting TBL-15° MoS_2_ electrocatalyst exhibits enhanced HER performance. It achieves a lower overpotential of 182 mV at 10 mA/cm^2^, a smaller Tafel slope of 63 mV/dec, and a charge-transfer resistance of 9.13 × 10^7^ Ω. These values are markedly improved compared with bilayer MoS_2_, which showed an overpotential of 322 mV, a Tafel slope of 202 mV/dec, and a resistance of 3.47 × 10^8^ Ω. These findings indicate that the effect of the twist angle influences interfacial charge transfer and reaction kinetics. Therefore, twist angle engineering provides an effective strategy for the design of high-performance 2D catalysts.

## Data Availability

The original contributions presented in this study are included in the article/Supplementary Material. Further inquiries can be directed to the corresponding author.

## Supplementary Materials

The following supporting information can be downloaded at https://www.mdpi.com/article/10.3390/ma19153211/s1. Figure S1: Potential offset of the SCE relative to a saturated Ag/AgCl electrode in 0.5 mol/L H_2_SO_4_ over 600 s; Figure S2: (a,b) Polar plots of parallel components of SHG in TBL-14° MoS_2_ and TBL-16° MoS_2_, respectively. Figure S3: (a) Dependence of the moiré period on the twist angle in bilayer MoS_2_. (b) SAED image of TBL-15° MoS_2_; Figure S4: Activation sequences are shown for (a) bilayer MoS_2_, (b) TBL-14° MoS_2_, (c) TBL-15° MoS_2_, and (d) TBL-16° MoS_2_; Figure S5: LSV curves from three devices are shown for (a) bilayer MoS_2_, (b) TBL-14° MoS_2_, (c) TBL-15° MoS_2_, and (d) TBL-16° MoS_2_; Figure S6: Overpotentials at current density of 10 mA/cm^2^ of the TBL-15° MoS_2_ in comparison to values reported previously for HER electrocatalytic nanodevices in acidic electrolytes [43,44,45,46,47,48,49,50,51,52].
