# Supplementary material for: Twist-Angle-Dependent Electrocatalytic Activation of Basal Plane MoS2 in Twisted Bilayer Structures
Source: Materials (Basel). 2026 Jul 28;19(15):3211. doi: 10.3390/ma19153211 (PMC13466550; doi:10.3390/ma19153211)
Supplement: Supplementary file 1 [file materials-19-03211-s001.zip › materials-4402436-supplementary.pdf]

Supplementary Materials for

Twist-Angle-Dependent Electrocatalytic Activation of Basal Plane MoS_2 in Twisted Bilayer Structures

Yuhang Chen [1], Huanbo Zhang [1], Hang Lu[1], Xiongfeng Li[1], Zhenyao Huang[1], and Mengyu Yan [1,2,*]

[1] State Key Laboratory of Advanced Technology for Materials Synthesis and Processing, School of Materials Science and Engineering, Wuhan University of Technology, Wuhan 430070 Hubei, China; cyh344767@whut.edu.cn (Y.C.); WHUTzhb2201@whut.edu.cn (H.Z.); 348935@whut.edu.cn (H.L.); 297062@whut.edu.cn (X.L.); a2328881382@163.com (Z.H.)
[2] Foshan Xianhu Laboratory, Xianhu hydrogen Valley, Foshan 528200, China
* Correspondence: ymy@whut.edu.cn

Supporting Experimental Details and Figures

A SCE was used as the reference electrode for the HER measurements in 0.5 mol/L H_2SO_4. The calibration results showed that the potential difference between the SCE and the saturated Ag/AgCl reference electrode was approximately 0.047 V (Figure S1). This value remained stable during the 600 s test period. The potential can be converted into

$$E_{\text{V } vs.\text{ SCE}} = E_{\text{V } vs.\text{ Ag/AgCl}} + 0.047\ \text{V}$$

where $E_{\text{V } vs.\text{ SCE}}$ represented the converted potential using SCE as the reference electrode. $E_{\text{V } vs.\text{ Ag/AgCl}}$ represented the potential obtained by calibrating the SCE using a standard Ag/AgCl. The standard Ag/AgCl reference electrode for calibration was filled with saturated KCl solution. Its standard potential is 0.197 V *vs.* the standard hydrogen electrode (SHE) at 25 ℃. The corresponding relationship can be expressed as

$$E_{\text{V } vs.\text{ SHE}} = E_{\text{V } vs.\text{ SCE}} + 0.244\ \text{V}$$

where $E_{\text{V } vs.\text{ SHE}}$ represented the potential converted to *vs.* SHE. All potentials in this work ($E_{\text{V } vs.\text{ RHE}}$) were converted to the reversible hydrogen electrode (RHE) scale according to

$$E_{\text{V } vs.\text{ RHE}} = E_{\text{V } vs.\text{ SHE}} + 0.059 \times \text{pH}$$

The pH of 0.5 mol/L H_2SO_4 was taken as 0.3. Thus, the potentials measured versus SCE were

converted to the RHE scale using

$$E_{V\ vs.\ RHE} = E_{V\ vs.\ SCE} + 0.2617\ V$$

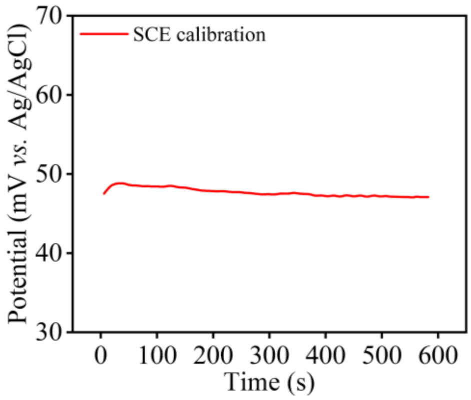

Figure S1. Potential offset of the SCE relative to a saturated Ag/AgCl electrode in 0.5 mol/L H_2SO_4 over 600 s.

The polarization-resolved second harmonic generation (SHG) patterns of the twisted bilayer MoS_2 samples are displayed in Figure S2. The SHG intensity distribution exhibited a clear sixfold rotational symmetry. A fit to the angular dependence gave a phase offset of 13.97° for the TBL-14° MoS_2 (Figure S2a). The fitted angular offset for the TBL-16° MoS_2 was 15.90° (Figure S2b). Both fitted values agree well with the respective twist angles.

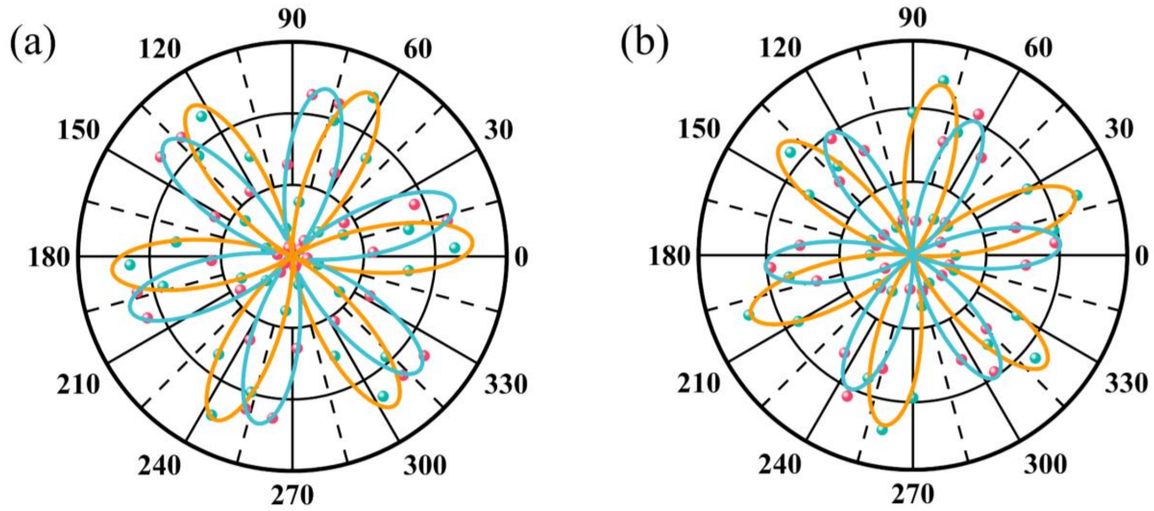

Figure S2. (a,b) Polar plots of parallel components of SHG in TBL-14° MoS_2 and TBL-16° MoS_2, respectively.

Figure S3a presents the calculated dependence of the moiré period on the twist angle for bilayer

MoS_2. The moiré period decreased rapidly with increasing twist angle and gradually approaches a constant value at larger angles. Based on this relationship, the twist angle of the fabricated sample was estimated from the diffraction measurement. Figure S3b showed the selected-area electron diffraction (SAED) pattern of the twisted bilayer MoS_2 (TBL-MoS_2). Two sets of hexagonal diffraction spots originating from the individual MoS_2 layers are clearly resolved. The angular separation between the corresponding diffraction vectors is measured to be approximately 15.48°, confirming the successful fabrication of a twisted bilayer structure with a twist angle close to 15°. The sharp and well-defined diffraction spots indicate high crystallinity and preserved hexagonal lattice symmetry.

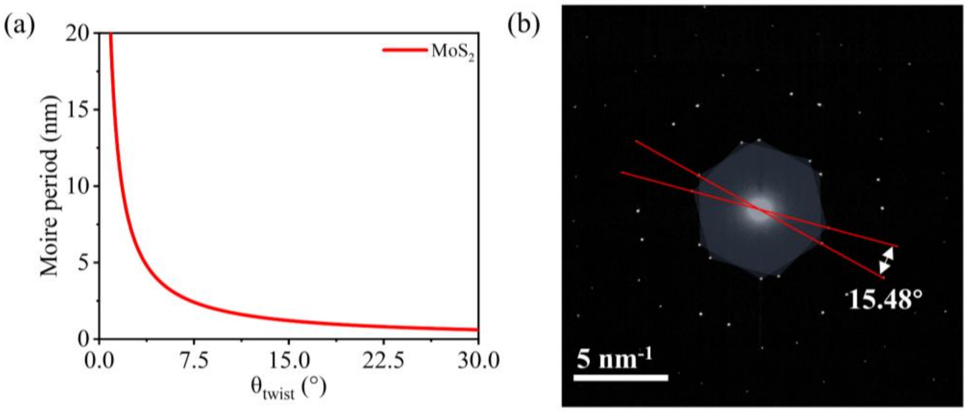

Figure S3. (a) Dependence of the moiré period on the twist angle in bilayer MoS_2. (b) SAED image of TBL-15° MoS_2.

Cyclic voltammetry (CV) activations were performed on all samples before the LSV measurements, including bilayer MoS_2, TBL-14° MoS_2, TBL-15° MoS_2, and TBL-16° MoS_2 (Figure S4a-d). During the initial cycles, the cathodic current gradually increased before reaching a stable response. This behavior is attributed to the electrochemical activation of the exposed surface, during which improved electrolyte wetting and removed the surface contamination.

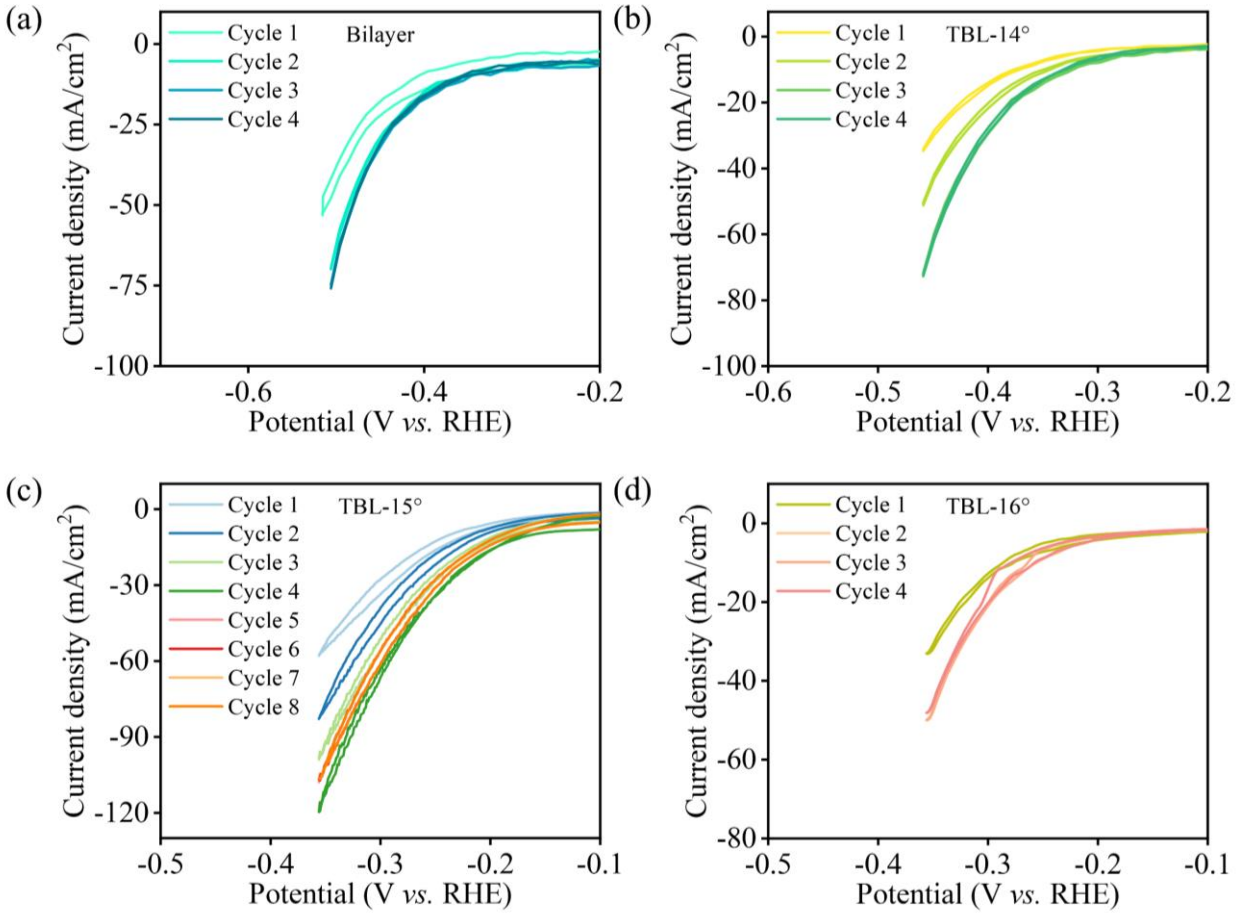

Figure S4. Activation sequences are shown for (a) bilayer MoS_2, (b) TBL-14° MoS_2, (c) TBL-15° MoS_2, and (d) TBL-16° MoS_2.

Repeated HER measurements were performed for each sample class, including bilayer MoS_2, TBL-14° MoS_2, TBL-15° MoS_2, and TBL-16° MoS_2 (Figure S5a-d). Three independent samples were measured for each group. The linear sweep voltammetry (LSV) curves showed similar onset behavior and comparable current responses within the same sample class. This confirms the reproducibility of the electrochemical measurements.

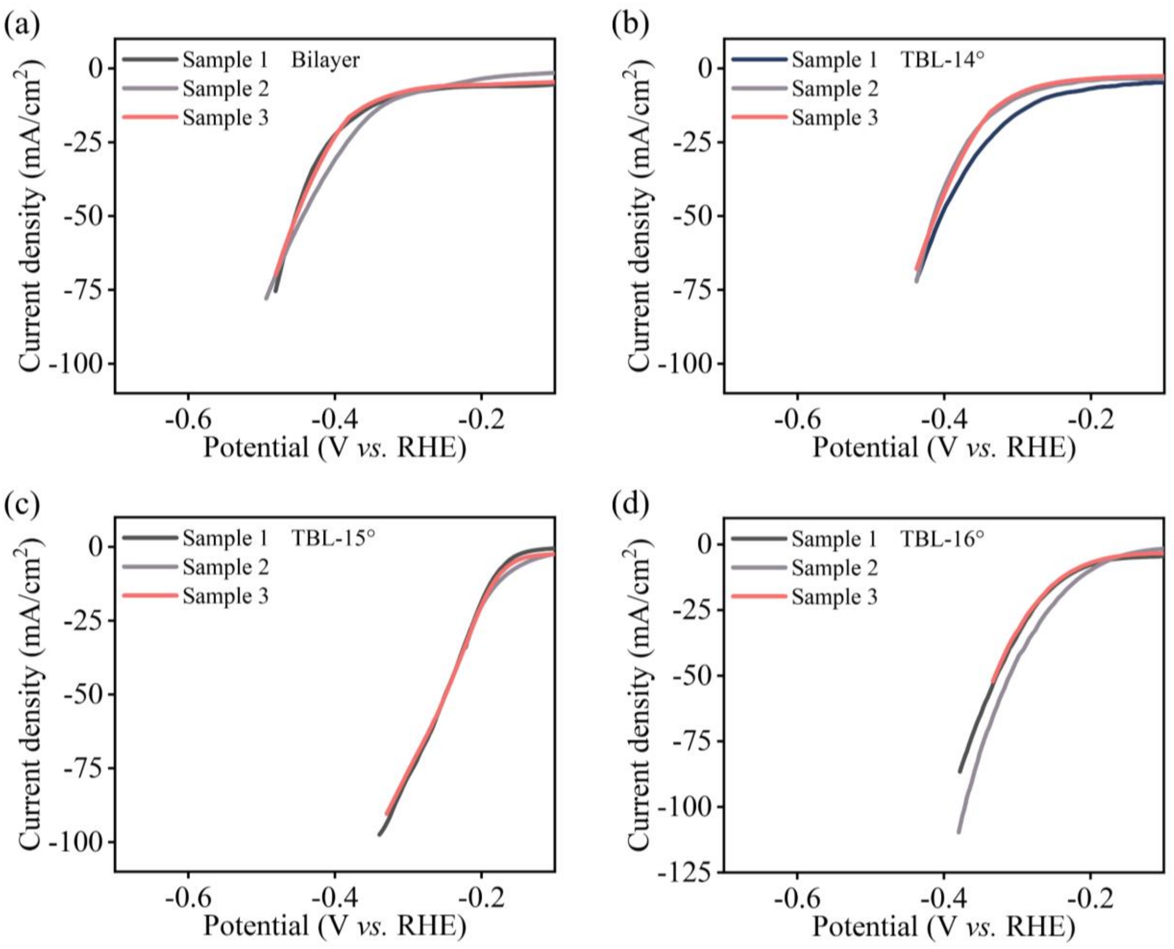

Figure S5. LSV curves from three devices are shown for (a) bilayer MoS_2, (b) TBL-14° MoS_2, (c) TBL-15° MoS_2, and (d) TBL-16° MoS_2.

The hydrogen evolution reaction (HER) performance of MoS_2 in 0.5 mol/L H_2SO_4 is compared with other reported catalysts (Figure S6). The results demonstrate that the MoS_2-based catalyst in this study exhibits competitive overpotentials and Tafel slopes compared to various other materials, including different MoS_2 phases and other MoS_2-based composites.

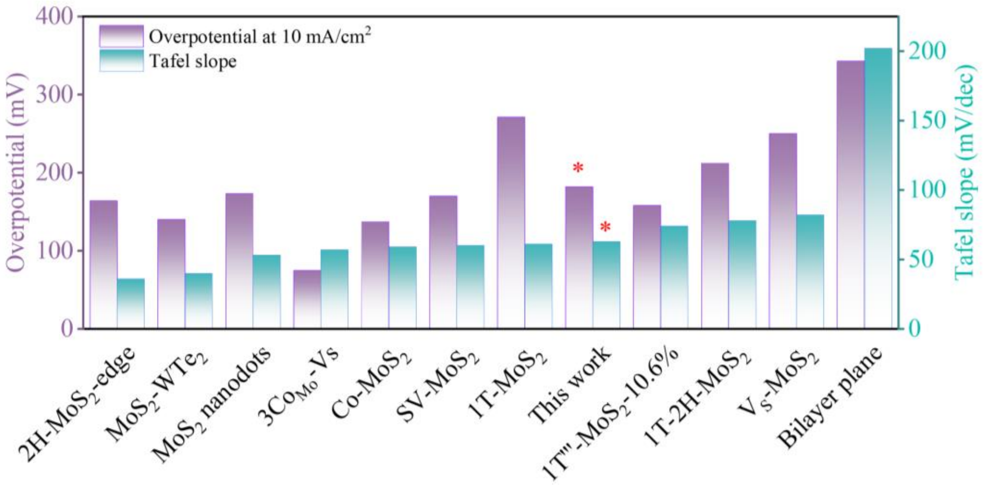

Figure S6. Overpotentials at current density of 10 mA/cm² of the TBL-15° MoS_2 in comparison to values reported previously for HER electrocatalytic nanodevices in acidic electrolytes [1-10].

References

[1] Li, H.; Tsai, C.; Koh, A. L.; Cai, L.; Contryman, A. W.; Fragapane, A. H.; Zhao, J.; Han, H. S.; Manoharan, H. C.; Abild-Pedersen, F. Activating and Optimizing MoS_2 Basal Planes for Hydrogen Evolution through the Formation of Strained Sulphur Vacancies. *Nat. Mater.* **2016**, *15* (1), 48–53. DOI: 10.1038/nmat4465.

[2] Benson, E. E.; Zhang, H.; Schuman, S. A.; Nanayakkara, S. U.; Bronstein, N. D.; Ferrere, S.; Blackburn, J. L.; Miller, E. M. Balancing the Hydrogen Evolution Reaction, Surface Energetics, and Stability of Metallic MoS_2 Nanosheets via Covalent Functionalization. *J. Am. Chem. Soc.* **2018**, *140* (1), 441–450. DOI: 10.1021/jacs.7b11242.

[3] Guo, X.; Song, E.; Zhao, W.; Xu, S.; Zhao, W.; Lei, Y.; Fang, Y.; Liu, J.; Huang, F. Charge Self-Regulation in 1T'''-MoS_2 Structure with Rich S Vacancies for Enhanced Hydrogen Evolution Activity. *Nat. Commun.* **2022**, *13* (1), 5954. DOI: 10.1038/s41467-022-33636-8.

[4] Hong, Z.; Hong, W.; Wang, B.; Cai, Q.; He, X.; Liu, W. Stable 1T–2H MoS_2 Heterostructures for Efficient Electrocatalytic Hydrogen Evolution. *Chem. Eng. J.* **2023**, *460*, 141858. DOI: 10.1016/j.cej.2023.141858.

[5] Takahashi, Y.; Kobayashi, Y.; Wang, Z.; Ito, Y.; Ota, M.; Ida, H.; Kumatani, A.; Miyazawa, K.; Fujita, T.; Shiku, H. High-Resolution Electrochemical Mapping of the Hydrogen Evolution Reaction on Transition-Metal Dichalcogenide Nanosheets. *Angew. Chem. Int. Ed.* **2020**, *59* (9), 3601–3608. DOI: 10.1002/anie.201912863.

[6] Zhang, J.; Wu, J.; Guo, H.; Chen, W.; Yuan, J.; Martinez, U.; Gupta, G.; Mohite, A.; Ajayan, P. M.; Lou, J. Unveiling Active Sites for the Hydrogen Evolution Reaction on Monolayer MoS_2. *Adv. Mater.* **2017**, *29* (42), 1701955. DOI: 10.1002/adma.201701955.

[7] Zhou, Y.; Pondick, J. V.; Silva, J. L.; Woods, J. M.; Hynek, D. J.; Matthews, G.; Shen, X.; Feng, Q.; Liu, W.; Lu, Z. Unveiling the Interfacial Effects for Enhanced Hydrogen Evolution Reaction on MoS_2/WTe_2 Hybrid Structures. *Small* **2019**, *15* (19), 1900078. DOI: 10.1002/smll.201900078.

[8] Tan, C.; Luo, Z.; Chaturvedi, A.; Cai, Y.; Du, Y.; Gong, Y.; Huang, Y.; Lai, Z.; Zhang, X.; Zheng, L.

Preparation of High-Percentage 1T-Phase Transition Metal Dichalcogenide Nanodots for Electrochemical Hydrogen Evolution. *Adv. Mater.* **2018**, *30* (9), 1705509. DOI: 10.1002/adma.201705509.

[9] Zhou, Y.; Zhang, J.; Song, E.; Lin, J.; Zhou, J.; Suenaga, K.; Zhou, W.; Liu, Z.; Liu, J.; Lou, J. Enhanced performance of in-plane transition metal dichalcogenides monolayers by configuring local atomic structures. *Nat. Commun.* **2020**, *11* (1), 2253. DOI: 10.1038/s41467-020-16111-0.

[10] Duan, H.; Wang, C.; Li, G.; Tan, H.; Hu, W.; Cai, L.; Liu, W.; Li, N.; Ji, Q.; Wang, Y. Single-Atom-Layer Catalysis in a MoS_2 Monolayer Activated by Long-Range Ferromagnetism for the Hydrogen Evolution Reaction: Beyond Single-Atom Catalysis. *Angew. Chem. Int. Ed.* **2021**, *133* (13), 7251–7258. DOI: 10.1002/anie.202014968.
